# Optimizing the Targeting of Mouse Parvovirus 1 to Murine Melanoma Selects for Recombinant Genomes and Novel Mutations in the Viral Capsid Gene

**DOI:** 10.3390/v10020054

**Published:** 2018-01-30

**Authors:** Matthew Marr, Anthony D’Abramo, Nikea Pittman, Mavis Agbandje-McKenna, Susan Cotmore, Peter Tattersall

**Affiliations:** 1 Department of Laboratory Medicine, Yale University Medical School, New Haven, CT 06520, USA; matthew.joseph.marr@gmail.com (M.M.); anthony.dabramo@yale.edu (A.D.); susan.cotmore@yale.edu (S.F.C.); 2 Department of Biochemistry and Molecular Biology, University of Florida, Gainesville, FL 32611, USA; nikea@ad.ufl.edu (N.P.); mckenna@ufl.edu (M.A.-M.); 3 Center for Structural Biology, The McKnight Brain Institute, University of Florida, Gainesville, FL 32611, USA; 4 Department of Genetics, Yale University Medical School, New Haven, CT 06510, USA

**Keywords:** protoparvovirus, oncotropic, melanoma, targeting, selection, recombination, mutation, enhancement, chimera, structure

## Abstract

Combining virus-enhanced immunogenicity with direct delivery of immunomodulatory molecules would represent a novel treatment modality for melanoma, and would require development of new viral vectors capable of targeting melanoma cells preferentially. Here we explore the use of rodent protoparvoviruses targeting cells of the murine melanoma model B16F10. An uncloned stock of mouse parvovirus 1 (MPV1) showed some efficacy, which was substantially enhanced following serial passage in the target cell. Molecular cloning of the genes of both starter and selected virus pools revealed considerable sequence diversity. Chimera analysis mapped the majority of the improved infectivity to the product of the major coat protein gene, *VP2*, in which linked blocks of amino acid changes and one or other of two apparently spontaneous mutations were selected. Intragenic chimeras showed that these represented separable components, both contributing to enhanced infection. Comparison of biochemical parameters of infection by clonal viruses indicated that the enhancement due to changes in VP2 operates after the virus has bound to the cell surface and penetrated into the cell. Construction of an in silico homology model for MPV1 allowed placement of these changes within the capsid shell, and revealed aspects of the capsid involved in infection initiation that had not been previously recognized.

## 1. Introduction

While melanoma is less common than other types of skin cancer, it causes 75% of all skin cancer-related deaths, with an expected incidence of >91,000 new diagnoses, and close to 10,000 deaths in the US during 2018 [1]. Unlike most cancers, its incidence is rising, and, until recently, the disease has resisted all treatment modalities. Despite initially poor responses to immunotherapy regimens [2,3], recent advances in such treatments, particularly adoptive transfer of tumor-infiltrating lymphocytes [4,5], and immunotherapy using antibodies to modulate immune checkpoints [6,7,8,9,10], have been increasingly successful, and indicate that shifting the balance of tumor-specific immune regulation in favor of cytotoxic T cell (CTL) activation, rather than T_reg_-mediated suppression, could achieve durable remission [11]. One way this might be further enhanced would be to exploit the induction of innate host responses to viral infection as an adjuvant to immunotherapy. With the use of appropriately targeted vectors, therapeutic immunomodulatory molecules could be generated at point sources within the tumor microenvironment, and thus, ameliorate the often severe side effects of their systemic application.

The idea of using the lytic activity of viruses to target cancer cells for destruction is not new. In the early 1950s, Moore and colleagues [12] pioneered studies on viruses with a predilection for tumor cells over their normal counterparts, and coined the phrase “oncolytic virus” to describe such agents. Interest in this approach has intensified recently, mainly driven by new understanding, at the molecular level, of the differences between transformed and normal cells, and the ways in which viral regulatory circuits intersect with these cellular pathways. Herpesvirus [13,14,15], adenovirus [16], Newcastle disease virus [17,18,19], reovirus [20], vesicular stomatitis virus [21], poxviruses [22], and rodent parvoviruses [23,24] have been evaluated as oncolytic agents against many cancer types, including melanoma, either as unmodified infectious virus, attenuated mutants, or vectors.

The rodent parvoviruses are members of the genus *Protoparvovirus* in the family *Parvoviridae*, among the smallest known viruses. They contain a unique linear single-stranded DNA genome, ~5 kb in length, which is flanked by small structured hairpin telomeres, and packaged into a rugged icosahedral (*T* = 1) protein capsid, 260–280 Å in diameter [25]. Inevitably, such tiny viruses are genetically constrained, with just two gene cassettes: an NS gene encoding the replication initiator endonuclease, and a small number of auxiliary proteins essential for infection of cells of their natural host species, and a VP gene encoding size variants of a single structural protein [26]. Although productive replication of some parvoviruses, most notably the adeno-associated viruses (AAVs), relies on coinfection with a helper virus from a range of double-stranded DNA virus families, most viruses in the *Parvoviridae*, and all known members of the genus *Protoparvovirus*, are capable of helper-independent, or “autonomous” replication. However, protoparvoviruses lack an intrinsic process for initiating the conversion of their single-stranded genome into double-stranded DNA, and until the cell progresses through S phase, the genome appears to remain sequestered in the capsid and incapable of transcription. Unlike other DNA viruses, these viruses do not possess any early-acting ancillary proteins that can induce non-cycling host cells to enter S phase, so they must wait until the cell prepares to enter the cell cycle, usually in response to exogenous growth signals [27]. Thus, they are inherently wired to replicate best in rapidly-dividing cell populations, making them particularly suited for targeting malignancy. However, the mechanism(s) underlying the inherent oncotropism of parvoviruses are more complex than just this S phase requirement, which can be met by any dividing cell, and current research indicates that normal human cells are able to suppress rodent parvoviral infection, while transformed cells lose that ability. In the family *Parvoviridae*, species *Rodent protoparvovirus 1* includes a number of distinct viruses that productively target specific cell types from an array of rodent species. However, while unable to infect normal human cells, several of these viruses can also infect and exhibit potent oncolytic activity against various types of transformed human cells. This species includes two murine viruses, the prototype strain of Minute Virus of Mice (MVMp) and Mouse Parvovirus 1 (MPV1), and the orphan virus, LuIII. We have previously shown that LuIII can infect and kill many primary human melanoma cell lines, a property that mapped to the LuIII capsid gene [28].

It is becoming increasingly apparent that a major indicator of efficacy for oncolytic viruses is the induction of an anti-tumor immune response [23,29,30,31]. Establishing whether transduction of melanoma cells with parvoviral vectors can enhance host immune responses to melanoma tumor antigens will require a tractable syngeneic animal model, for which we chose the well-characterized B16F10 transplantable mouse melanoma [32]. In order to identify the optimal virus on which to base such vectors, we first screened B16F10 cells for their susceptibility to infection by members of a panel of rodent protoparvoviruses. The optimal virus from this screen was then serially passaged in B16F10 cells, to select for variants enhanced in their ability to initiate infection. Subsequent analysis revealed that much of the enhanced infectivity selected by this process was a property of the *VP2* gene, suggesting that enhanced transduction of these murine melanoma cells could be achieved by packaging vector backbones in capsids comprising one of the selected *VP2* gene sequences.

## 2. Materials and Methods

### 2.1. Viruses, Cells, and Selection of MPV1 on B16F10 Cells

Viral stocks of MVMp (GenBank accession # J02275) and LuIII (GenBank accession # M81888) were generated by transfecting plasmid DNA into human 293T cells, as previously described [28,33]. An uncloned virus stock, denoted MPV1, originally isolated in 1994 [34], was kindly provided by Dr. Susan Compton, Department of Comparative Medicine, Yale School of Medicine. This stock was expanded by passage in SV40-transformed human NB324K cells [35]. B16F10 murine melanoma cells were the kind gift of Dr. Mark Mamula, Rheumatology Division, Department of Internal Medicine, Yale School of Medicine, New Haven, CT, USA. For the selection process, sub-confluent B16F10 cells were infected with this stock at low multiplicity. When the monolayer showed extensive cytopathic effect, usually after ~3 days, the cells were harvested by scraping into the medium, spun down, and resuspended in 50 mM Tris, 0.5 mM EDTA pH 8.7 (TE8.7 buffer). The sample was rapidly frozen in dry ice and thawed on ice three times to release virus particles from the cells. After centrifugation, an aliquot representing 0.2–1.0% of the supernatant containing the virus was applied to a fresh culture of B16F10 for re-infection. This process was repeated for a total of five times. The resulting virus pool is denoted MPV1p5 for the five passages it underwent in B16F10 cells.

### 2.2. Viral Sequencing

Fragments of the viral genome were amplified by PCR. Primers were designed to span the start of non-structural protein 1, *NS1*, 5′-CCCGTTTAAACCAAGGCGCGAAAAGGAAGTGGGCG-3- to the *VP1*:*VP2* junction, 5′-CCGAGCTCTGCTGTGCAGCAAGAGAAGCACGTTTTTT-3-, incorporating PmeI and SacI sites, respectively (underlined), and from the start of VP2, 5′-CAGCAGAGCTCTCAGACAATGAGTGATG-3G to the end of the 3’ untranslated region (3′UTR), 5’-ATAGACAGTAGTCTTGGTTAG-3’, incorporating SacI and PshAI sites, respectively (underlined). An additional reverse primer spanning the end of *VP2*, 5′-TGGTTAATTAATAAGTATTTCTAGCA

ACAG-3′, incorporating a PacI site (underlined), was designed to allow construction of the first set of founder infectious clones, because the conserved PshAI site in the NS gene complicated construction of infectious clones extending through the 3′UTR. This strategy allowed the subsequent direct substitution of this region for the equivalent sequence block within the infectious clone of MVMp, using a SacI site engineered at the C-terminus of the VP1sr, just upstream of the *VP2* start site in the MVMp infectious clone. Creation of a set of infectious clones containing the 3′UTR of MPV1, rather than that of MVMp, necessitated eliminating a PacI site in the middle of the MPV1 3′UTR by changing its sequence from TTAATTAA to TTAATTAT. This allowed substitution of the 3′UTR between PacI and PshAI sites in the recipient infectious clones.

The parental MPV1 and MPV1p5 viruses were denatured at 95 °C for 10 min, followed by PCR using high fidelity Phusion polymerase. A-tailing of the PCR product was performed with Taq DNA polymerase (New England Biolabs, Ipswich, MA, USA). The product was incorporated into either Topo pCR 2.1 (Thermo Scientific, Waltham, MA, USA) or NEB pMiniT vectors, and transformed into *E. coli*. All reactions and procedures were carried out according to the manufacturers’ instructions. Plasmids were then sequenced by using proprietary primers for the respective cloning vectors at Yale University’s Keck Sequencing Center.

### 2.3. Viral Subcloning, Recovery, Purification, and Quantification

The *NS1*/*NS2/VP*1 sequence was isolated by digestion with PmeI and SacI restriction enzymes. The *VP2* sequence was isolated by digestion with SacI and PacI. Chimeras were formed by ligating the appropriate fragments and the MVMp infectious clone backbone with NEB quick ligase, and transforming into Agilent SURE-2 supercompetent cells. Correct terminal hairpin lengths were confirmed by digestion with AflIII, followed by agarose gel electrophoresis. Chimeric viruses were recovered in 293T cells following transfection with polyethylene imine (PEI), as previously described [33,36,37]. Briefly, 2 × 10^6^ 293T cells were plated in 10 cm dishes the day before transfection. Each dish was treated with the transfection mixture of 4 μg infectious clone DNA, 6 μg pXX6-80 helper plasmid [38], and 25 μL PEI. Cells were harvested after 72 h, extracted by serial freeze/thaw cycles, cleared by centrifugation, purified on iodixanol step gradients, and quantified by alkaline agarose gel electrophoresis and Southern blotting, as described previously [33,39,40].

### 2.4. Analysis of Viral Infection Efficiency

The infectivity of the viruses and chimeras was assessed using flow cytometry, as described previously [28]. Six-well plates were seeded with 2 × 10^5^ B16F10 cells per well in DMEM media supplemented with 5% fetal bovine serum (FBS). One day after seeding, the cells were infected with virus in 1 mL of DMEM containing 1% FBS. After 4 h, 1 mL of DMEM containing 9% FBS was added, and the cells were incubated for a further 20 h, before being harvested with trypsin, fixed in 1% paraformaldehyde, permeabilized with 0.5% Triton, blocked in 10% normal goat serum in PBS, stained with mouse α-NS1 mAb (CE10B10 [41]) in the blocking solution, and incubated with BioLegend goat α-mouse IgG DyLight 488 secondary antibody. Flow cytometry was performed on a MACSQuant Analyzer 10 (Miltenyi Biotec Inc., Auburn, CA, USA), set to record 10,000 events, using gating values established with infected cells, prepared as described above, but omitting incubation with the primary antibody. The positive gate was set so that ≤0.5% of the uninfected, stained control cells were positive. Results are expressed in histogram form with error bars that represent the standard deviation of the mean for duplicate infections.

### 2.5. Analysis of Viral Gene Expression and DNA Replication

B16F10 cells were seeded in 6-well plates, as described above. Growth medium was removed and replaced with 0.5 mL of DMEM containing 1% FBS and 20 mM HEPES (4-(2-hydroxyethyl)-1-piperazineethanesulfonic acid) buffer, pH 7.3, containing virus at the appropriate concentration. Plates were rocked every 30 min for 4 h, viral inocula were removed, and the cultures incubated for a further 2 h with fresh medium containing 0.04 units per mL of neuraminidase (*Clostridium perfringens*, type V; Sigma, St. Louis, MO, USA) to remove surface virus, after which the medium was changed once more with medium containing neuraminidase to prevent reinfection. For gene expression analysis, cells were lysed directly at 24 h, and their protein content quantified with a Pierce BCA Protein Assay Kit (Thermo Scientific, Waltham, MA, USA), as previously described [42]. Fifty microgram aliquots (with 100 mM DTT and loading dye) were loaded in a 4–20% Mini-Protean TGX Gel (Bio-Rad, Hercules, CA, USA), and transferred to a Hybond ECL membrane (GE Healthcare, Little Chalfont, UK). Membranes were blocked, incubated with a rabbit antibody directed against MVM NS1/2 N-terminal peptide for the non-structural proteins, or a rabbit antibody directed against denatured MVMp capsids [43,44], and developed using an ECL system, according to the manufacturer’s instructions (Amersham, Uppsala, Sweden). For DNA replication analysis, infected cells were harvested at 6 and 24 h, DNA extracted from cell pellets, separated by neutral agarose gel electrophoresis, and detected by Southern blot analysis, as previously described [36,45].

### 2.6. Modeling of MPV1 Structures

A 3D model of MPV1p5-Bv1 VP2 was generated using the online SWISSMODEL automated modeling pipeline (http://swissmodel.expasy.org/workspace/index.php?func=modelling_simple1) with the coordinates of the MVMp VP2 structure (PDB ID: 1Z14; [46]) supplied as a template [47]. The VP2 model extended from N-terminal residue 47 to the last C-terminal residue, 587. This VP2 coordinate file was used to generate a 60-mer by *T* = 1 icosahedral matrix multiplication using the Oligomer Generator subroutine in the ViperDB online server (http://viperdb.scripps.edu/oligomer_multi.php) [48] to help visualize the location of the four variant residues, R54, S57, M70, and N348 in the MPV1p5-Bv1 variant compared to the parental clone, MPV1-Mv1. This enabled the analysis of potential dimer, trimer, and pentameric symmetry-related interactions in the program COOT [49]. The VP2 model was also used to generate a 2D “Roadmap” image, depicting the surface exposed residues and highlighting the positions of the amino acid differences between MPV1p5-Bv1 and the parent clone, within a viral asymmetric unit, using the RIVEM program [50]. The PyMol molecular graphics program [51] was used to produce a cartoon image of the VP2 monomer, on which variant amino acid positions were visualized.

### 2.7. GenBank Accession Numbers

Viral genome and protein sequences were derived from those deposited in GenBank under the following accession numbers: MPV1—U12469; MPV1b—U24253; MPV1c—U34254; MPV1d (MPV UT)—AB234204; MPV1e—DQ898166; MPV1-UL1—KC249519; MPV1-UL4—KC249522; MPV1-UL5—KC249523; MPV1-UL6—KC249524; MPV2—DQ196319; and MPV2 UL3—KC249521.

## 3. Results

### 3.1. Screening for Viruses That Target Murine Melanoma

**Figure 1:**
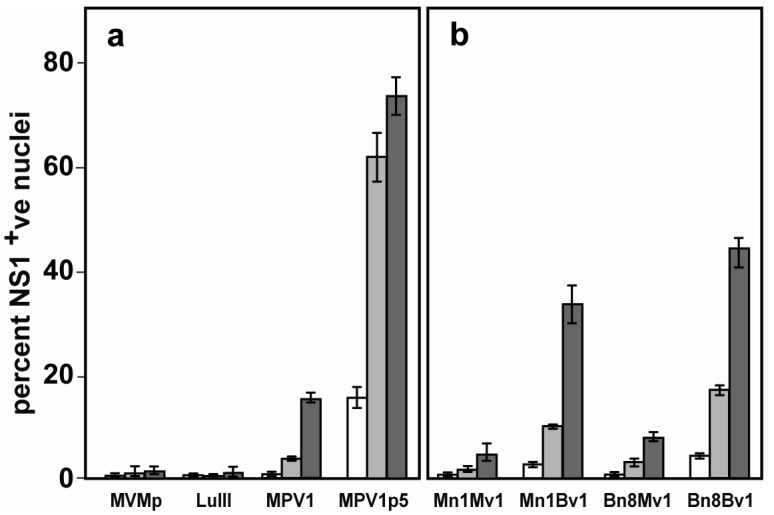
Infection of B16F10 cells with individual protoparvovirus strains. B16F10 cells were infected, at 5-fold increasing multiplicities, with panel (**a**) MVMp, LuIII, and uncloned Mouse Parvovirus 1 (MPV1) stocks, and panel (**b**) viruses derived from MPV1 founder infectious clones. Viruses derived from the founder clones, described in Section 3.2 below, are named for the originating virus stock, “M” for the parental MPV1 or “B” for the B16F10-passaged virus MPV1p5, and “n” for the NS/VP1 region or “v” for the *VP2* region, followed by the number of the sample from which it was derived, as shown in Figure 2 and Figure 3 below. White bars = 200, light grey bars = 1000 and dark grey bars = 5000 genome equivalents per cell. Twenty-four hours after infection, cells were fixed, permeabilized, stained for NS1, and analyzed by flow cytometry, as described in the Methods section. Error bars represent the standard deviation for duplicate infections.

B16F10 cells in culture were screened with a seven member parvovirus panel, plus five additional strains of MVM, in a multiwell dose response assay, as previously described [52]. Significantly, LuIII, which is melanomatropic in human cells [28], showed no activity against B16F10, and only MPV1 showed significant, albeit limited, ability to infect these cells. While we do not yet understand the molecular basis of the tropism displayed by rodent protoparvoviruses for human or murine melanomas, there is no a priori reason to believe that this property is optimal for any one virus strain. Since parvoviral replication yields a single nucleotide mutation for every 10^4^ bases [45,53,54], with a 5000 bp genome, on average, every other viral genome should carry a single base mutation. Although most of these will be either silent or deleterious, selection on B16F10 cells should yield quasi-species of mutant MPV1 parvoviruses with enhanced levels of melanoma infectivity. Since the MPV1 infection was not robust, the uncloned stock was serially passaged five times in B16F10 cells. The uncloned pool of serially passaged virus was purified, quantitated, and assayed versus MVMp, LuIII, and its parental pool, in a flow cytometric initiation assay in which infection rates were determined at 24 h after infection at escalating input multiplicities. As shown in Figure 1a, this assay confirmed the low infection rates achieved by MVMp and LuIII, and the somewhat greater initiation efficiency of MPV. However, the serially passaged virus pool, MPV1p5, initiates infection in B16F10 cells about five times more efficiently than genome-matched doses of the parental MPV1 virus pool, and at least 10-fold more than either MVMp or LuIII.

### 3.2. Genetic Analysis of the Parental and Selected Viruses

Recovery of viral genome segments was performed by PCR amplification directly from uncloned pools, and as we have not been able to reliably isolate PCR products spanning the whole 5 kb viral chromosome, we recovered viral sequences in approximately equal halves of the genome. Since the human melanoma targeting potential of LuIII was mapped to the *VP2* gene [28], we examined this region first.

**Figure 2:**
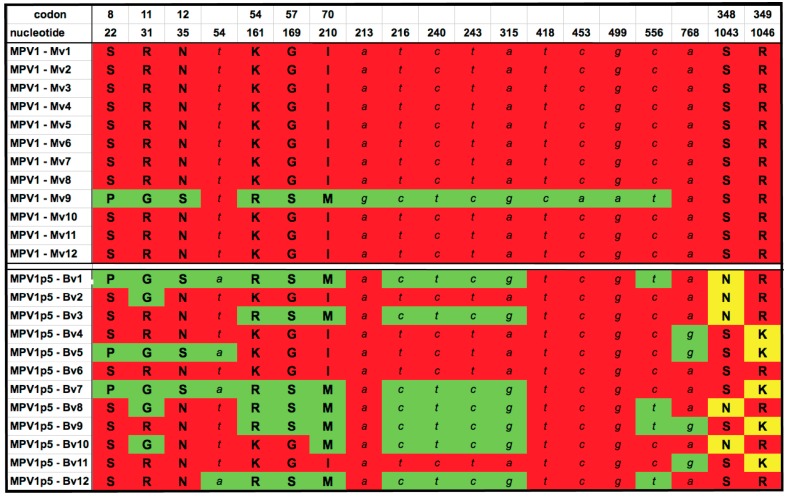
Analysis of mutations found in the *VP2* region. Twelve clones from the MPV1 starter pool and twelve clones from MPV1p5 were sequenced and compared to one another. Synonymous nucleotide differences are indicated in italicized lower case, while nucleotide changes that resulted in amino acid substitutions are denoted by the single-letter amino acid code for the encoded residue in upper case bold. Both synonymous and non-synonymous mutations found in more than one PCR clone are included. Clones are named for the originating virus stock, “M” for parental and “B” for B16F10-passaged virus, and “v” for the *VP2* region, followed by the sample number. Red and green shading denote sequences conforming to the predominant sequences of MPV1 parental and MPV1p5 *VP2* genes, respectively. The two mutant residues that are unique to MPV1p5 *VP2* clones are shaded in yellow.

The sequences of the recovered clones differed at 17 amino acid residues from the sequence deposited in GenBank for the original MPV1 [34]. Of these 17 changes, only the 8 that differed between clones of the parental and passaged viruses are shown in Figure 2. These 8 changes occurred within the N-terminal 350 codons of the 587 amino acid protein, while the remaining 9 residues were invariant between all 24 clones sequenced. All but one of these were located in the C-terminal third of the molecule, and all nine were omitted from this figure for the sake of simplicity. Figure 2 shows that the great majority of clones derived from the MPV1 parental strain are identical to one another, indeed, only one of the 12 sequenced parental clones, MPV-Mv9, differed from the consensus sequence, and conformed more closely to the GenBank MPV1 sequence. However, 11 clones isolated from the passaged virus exhibited varying combinations of 8 mutations in the *VP2* region that matched, to varying extents, the Mv9 outlier sequence among the parental clones. Three of these amino acid changes, S8P, R11G and N12S, are located in the VP2 N-terminal sequence that is extruded through the five-fold pore upon packaging of the viral genome [55]. Notable among the other changes are the two most C-terminal mutations, S348N and R349K, because most of the MPV1p5 clones appear to have one of these mutations, but never both. They are also unique, because they were the only mutations in the selected viruses not found in any of the nine MPV1 *VP2* sequences available in GenBank, as discussed below. In addition, 11 synonymous changes from the MPV1 *VP2* gene sequence in GenBank occurred within the 24 *VP2* clones, but were not located in any regions of known importance at the DNA sequence level.

**Figure 3:**
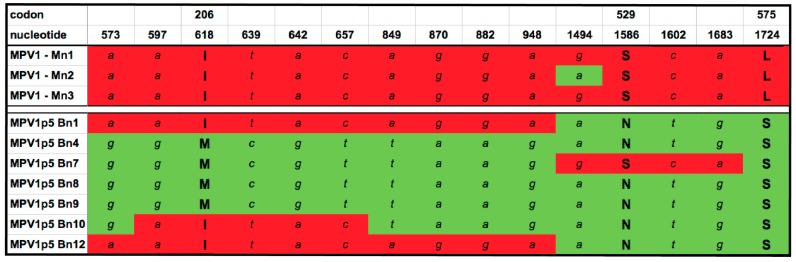
Analysis of mutations found in the *NS*/*VP1* region. Three clones from the starter MPV1 pool and seven clones from MPV1p5 were sequenced and compared to one another. Both synonymous and non-synonymous mutations found in more than one PCR clone are included. As before, clones are named for the originating virus stock, “M” for parental and “B” for B16F10-passaged virus, and “n” for the *NS*/*VP1* region, followed by the sample number. NS2 and VP1sr protein sequences were identical between all MPV1 and MPV1p5 clones. Red and green shading denote sequences conforming to the predominant sequences of the MPV1 parental and MPV1p5 NS/VP1 clones, respectively.

**Figure 4:**
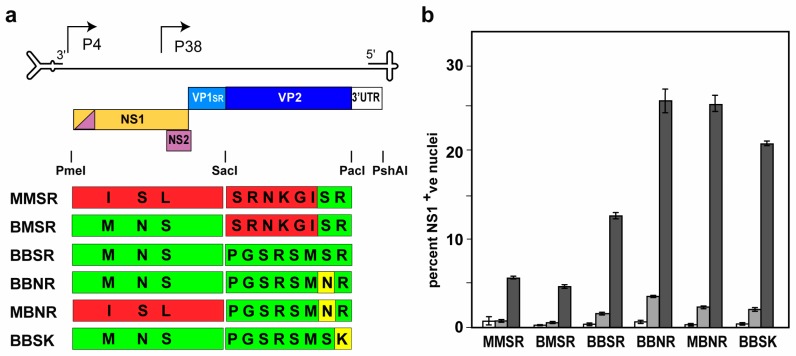
Construction and analysis of chimeric MPV1 and MPV1p5 viruses. In (**a**), the genetic strategy of the protoparvoviral genome is shown above as a bar diagram representing maps of the chimeric viruses created from clones described in Figure 2 and Figure 3. Chimeras were constructed by insertion of *NS/VP1* or *VP2* sequences between the PmeI and SacI, or SacI and PacI sites, respectively, in the MVMp backbone. For this set of chimeras, the MVMp 3′UTR was substituted with the MPV1 sequence between the PacI and PshAI sites in each construct, as described in the text. The colors in the chimera diagrams reflect those assigned to the appropriate sequence blocks in Figure 2 and Figure 3. (**b**) B16F10 cells were infected with the indicated viruses at 10-fold increasing multiplicities (white bars = 50, light grey bars = 500, and dark grey bars = 5000 genome equivalents per cell) and analyzed for NS1 expression by flow cytometry, as described for Figure 1. Each infection was performed in duplicate, and error bars represent the standard deviation. Naming is based on the origin of each fragment, M for MPV1 and B for MP1p5. The first letter denotes the origin of the *NS/VP1* fragment, the second letter denotes the *VP2* fragment, and the third and fourth letters denote the amino acids present at codons 348 and 349 in *VP2*.

The 3’UTR was identical in all MPV1 parental and MPV1p5 SacI to PshAI clones sequenced, and only differed from the MPV1 GenBank sequence by 3 out of 355 nucleotides. The only regions of the two virus pools that were not sequenced were the right- and left-end terminal hairpins, which tend to be conserved within the protoparvoviruses, and are difficult to clone and sequence. Half of the 34 PCR-derived clones analyzed in Figure 2 and Figure 3 below, contained one or more additional mutations that were unique to that clone, not appearing in any other clone or in any of the GenBank sequences. These were considered to be PCR errors, sequencing errors, or random unselected viral mutations. They were not included in the analyses shown in Figure 2, and were excluded from the genomes of the founder and chimeric infectious clones described below, in Figure 4a.

We then analyzed the left-half of the viral coding region, using a fragment that contained the *NS1*, *NS2*, and *VP1*-specific region (VP1sr) sequences, isolated as a ~2.7 kbp amplicon denoted *NS*/*VP1*, using the primers described in Section 2.2, incorporating unique restriction enzyme sites that facilitated the chimera constructions detailed below. Figure 3 shows the synonymous base changes, and predicted protein sequence differences among the *NS1* genes isolated from the MPV1 parental stock and the serially passaged virus. The *NS1* region contained one amino acid change from the original GenBank MPV1 sequence, L575S, that was found in all seven MPV1p5 clones, and two other mutations, I206M and S529N, that were found in a majority of the MPV1p5 NS/VP1 clones. All three of these non-synonymous *NS1* mutations occurred between the splice sites that give rise to *NS2*, therefore, the three known splice variants of this ancillary viral NS2 gene product are predicted to be identical between parental and selected viruses. Likewise, no predicted amino acid sequence variants between the parental and selected viruses were observed within the VP1sr.

Consistent with the *VP2* analysis in Figure 2, the majority of the non-synonymous mutations found in MPV1p5 are identical to the original MPV1 GenBank sequence, but differ from the parental MPV1 strain used as the starting material. Thirteen silent changes from the GenBank sequence were found within the left-end fragments, but were not located in any regions of known importance, such as the *NS1/NS2* splice sites, the various known elements of the P38 promoter, or the TATA box-proximal region of the P4 promoter.

### 3.3. Mapping the Element(s) That Enhance Growth in B16F10 Melanoma Cells

Using the sequencing data summarized in Figure 3, MPV1 and MPV1p5 founder infectious clones were constructed using Mn1 and Bn8 NS/VP1 regions, respectively, inserted between the PmeI to SacI sites in the MVMp infectious clone. Because a conserved PshAI site within the *NS1* region complicated the construction of clones, including the MPV1 3′UTR, the NS/VP1 constructs were combined with their cognate *VP2* genes, Mv1 and Bv1, respectively, by insertion between the SacI and PacI sites engineered at the N- and C-termini, respectively, of the *VP2* gene in the same MVMp background. Thus, the left- and right-end hairpin, and the 3’UTR in each of these founder clones are identical and derived from MVMp. Construction by serial insertion into the MVMp backbone allowed derivation of additional chimeric clones in which the MPV1 NS/VP1 region was paired with the MPV1p5 *VP2* gene, and vice versa. The resulting four clones, denoted Mn1Mv1, Bn8Bv1, Mn1Bv1, and Bn8Mv1, were recovered as viruses, and tested to determine whether they initiated infection similarly to the polyclonal stocks of MPV1 and MPV1p5, respectively, as shown in Figure 1.

As suggested by previous results with other protoparvoviruses [28,56], the component of the Bn8Bv1 founder clone most significant to its increased infection initiation in B16F10 cells is the *VP2* gene, which increases the infectivity of Mn1Mv1 from 5 to 34% in the chimeric Mn1Bv1 virus. The NS/VP1 region plays a relatively minor role in the evolution of MPV1p5, increasing the infectivity of the chimeric Mn1Bv1 virus from 34 to 45% in Bn8Bv1. Neither of the founder constructs performed as well as the polyclonal virus stock from which it was derived. At the highest titers of 5000 genomes per cell, Mn1Mv1 infected 5% of cells at 24 h, while MPV1 infected 16%. At the same input multiplicity, Bn8Bv1 infected 45% of cells, while the uncloned MPV1p5 pool infected 74%. Thus, there still remains a 2–3-fold difference between the polyclonal stocks of MPV1 and MPV1p5, and their equivalent versions cloned into the MVMp backbone. This suggests that there may be additional sequences in the MPV1 hairpins or 3′UTR that contribute to the ability of MPV1 to grow better than MVMp in B16F10 cells.

### 3.4. Determining the Contribution of the 3′UTR and the Unique VP2 Mutations

At 368 nucleotides, the 3′UTR of MVMp is 13 nucleotides longer than that of MPV1a, due to two insertions, one of 12 nucleotides plus a single nucleotide. The remaining 355 nucleotides align, except for 27 mismatches, scattered through the sequence. To test whether the additional host range element(s) discussed above resided in the 3′UTR, we re-constructed the founder and chimeric clones described above by inserting the MPV1 3′UTR into each, creating the clones MMSR, BMSR, BBNR, and MBNR, as shown in the top panel of Figure 4a. We used the BBNR backbone to construct a further pair of chimeras, also shown in Figure 4a, in which the region of the Bv1 sequence around codons 348 and 349 were exchanged, to create the parental sequence, S348; R349, to make BBSR, or to introduce the R349K mutation next to the wildtype S348 residue, to create BBSK.

### 3.5. Mapping the Enhancing Element(s) Using Chimeric Viruses

Figure 4b shows the analysis, by flow cytometric infection initiation assay, of viruses derived from the six constructs diagrammed in Figure 4a. Comparison of the results for MMSR and BMSR substantiate the previous finding that the changes in the *NS/VP1* region alone do not significantly increase the infectivity of the virus for B16F10 cells. Comparison of these with results obtained with BBSR and BBNR indicates that the block of changes that “revert” the VP2 coding sequence to match the MPV1 GenBank sequence, and the unique S348N mutation, each contribute about half of the ~5-fold increase between fully enhanced virus and its parent. Comparison of BBNR with MBNR confirm that the changes in the NS/VP1 region neither contribute significantly to, nor are required for, the 5-fold infectivity enhancement, which appears to be entirely a property of changes within the *VP2* gene. Switching the unique S348N mutation for its neighboring R349K change, as in BBSK, reduces the enhancement somewhat, although it is still significantly increased over that shown by BBSR. Comparison of the maximal initiation rate obtained at 5000 genomes per cell for BBNR or MBNR, with the rate observed for the uncloned MPV1p5 stock reported in Figure 1b, shows that the difference between them still persists at 2–3 fold. We take this to indicate that the 3′UTRs do not influence enhancement, and that the element(s) responsible for this further increase in infectivity for B16F10 cells likely resides in either or both of the terminal hairpins present in the uncloned viruses.

### 3.6. Biochemical Correlates of the Enhancement of Infectivity

To explore the biochemical mechanism(s) underlying the enhancement of infection initiation due only to the mutations in *VP2*, we set up infections at matched input multiplicities of virions generated from clones of either MMSR and MBNR, at 5000 genomes per cell (5K). In order to try to match the fraction of cells infected with these viruses, we also included an infection with MBNR at 1000 genomes per cell (1K).

**Figure 5:**
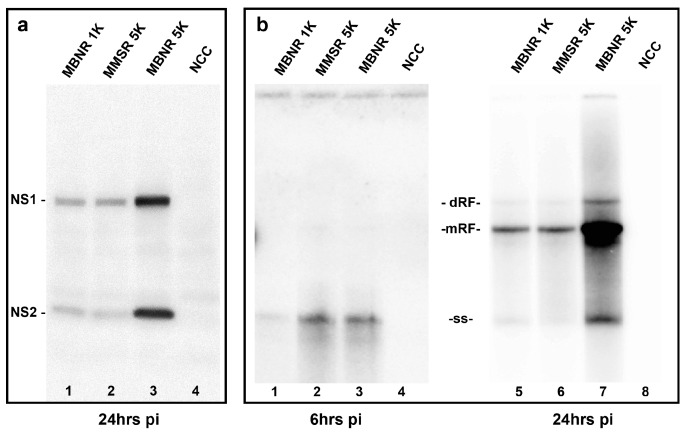
Comparison of non-structural gene expression and viral DNA accumulation. B16F10 cells were infected, as described in the Methods section, with MMSR at 5000 genomes per cell (5K), or with MBNR at 1000 (1K) or 5000 (5K) genomes per cell. (**a**) shows a Western blot of an SDS polyacrylamide gel of NS protein accumulation by 24 h after infection, probed with an antibody raised against the N-terminal region common to all NS1 and NS2 species; (**b**) shows a Southern blot of an agarose gel hybridized with a probe synthesized from the *NS1* sequence of MPV1. Intracellular viral DNA was extracted at 6 or 24 h after infection, as described in the Methods section. dRF, mRF and ss denote the migration positions for dimer replicative form, monomer replicative form, and single-stranded viral genomes, respectively. Lanes 1–4 were exposed for ~10 times longer than lanes 5–8.

As seen in Figure 5a, the MBNR 1K and MMSR 5K infections resulted in equal accumulations of NS1 and NS2, which correlated well with flow cytometry results that indicated 13% and 14% of cells in these two infections were NS1 positive. Consistent with this result, flow cytometric analysis showed that the median fluorescence index (MFI) for these two populations of infected cells were essentially identical. Under these experimental condition, the 5K infection with MBNR exhibited 38% NS1-positive cells, a 3-fold increase over the MMSR 5K infection. However, the increase in NS1 accumulation was several fold greater than expected, and this anomaly was also reflected in a 50% increase in the MFI over that of the 1K infection with the same virus. We suggest that this is due to individual cells infected at the higher multiplicity, initiating more viral transcription templates, and therefore, making more NS protein per infected cell.

The same phenomenon is seen for viral DNA species accumulated by 24 h after infection, where the MBNR 1K infection closely matches the MMSR 5K infection, while cells in the MBNR 5K infection resulted in disproportionately more viral DNA replication than expected, compared to cells infected with the same virus at 1K input., As expected, the accumulation of input viral genomes at six hours after infection closely followed the input multiplicity, as shown in Figure 5b, lanes 1–3, indicating that equal numbers of genomes became occluded in both the MMSR and MBNR 5K infections. Crucially, however, these occluded genomes gave rise to substantially different levels of viral NS protein expression and DNA replication, compare Figure 5b, lanes 2 and 3 with Figure 5a, lanes 2 and 3, and Figure 5b, lanes 6 and 7. This indicates that the enhancement of infectivity provided by the changes in VP2 sequence operates after the virus has bound to the cell surface, and penetrated into the interior of the cell.

## 4. Discussion

### 4.1. Targeted Evolution of the MPV1 Capsid

The initial objective of this study was to identify the most promising candidate rodent protoparvovirus to develop as a vector for use in the B16F10 murine melanoma model. Having done that, we were able to show that the targeting properties of this crude virus stock could be enhanced by serial passage in the target cell. The first surprise coming from this analysis was that the majority of sequences recovered from the starting virus did not conform to the sequence of the original MPV1 deposited in GenBank. Equally unexpected was the observation that genomes selected by passage in B16F10 cells tended to “revert” to the MPV1 GenBank sequence. These sequence changes appeared to have been inherited in blocks, suggesting the involvement of multiple recombination events. The recovery of one almost perfect match to the original MPV1 GenBank sequence out of the twelve parental clones implies that this subpopulation has been selected for during passage, and that the parental pool had been expanded in a host cell that selected the alternative population observed. Our original hypothesis was that the relatively high spontaneous mutation rate that the parvoviruses exhibit would generate novel amino acid substitutions that could be selected for, if they provided an improvement in essentially any step in the viral life cycle in B16F10 cells. Furthermore, re-assortment through recombination would allow selection of groups of advantageous mutations, leading to substantial increases in viral fitness in the melanoma host cell. That extensive recombination does occur during the expansion and serial passage of these viruses is strongly suggested by the appearance of apparently linked sequence blocks interspersed throughout the selected genomes, particularly in the *VP2* gene. The finding that alternative amino acid residues tend to be inherited in blocks makes it hard to determine whether individual mutations are enhancing, or adaptive, in that they allow a linked enhancing mutation to be selected by stabilizing any deleterious structural change in the capsid induced by the enhancing mutation. On the other hand, they may just be fortuitously linked and contribute nothing to the selected phenotype. Further chimera mapping will be required to address this question definitively.

**Figure 6:**
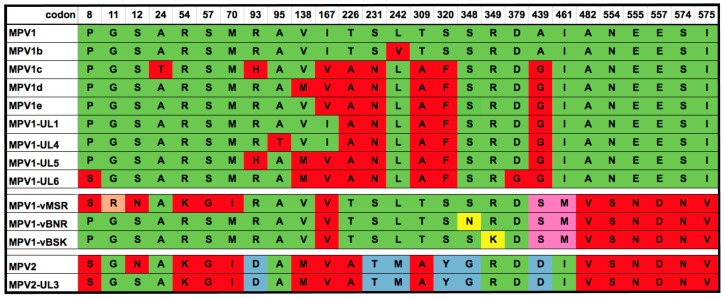
Alignment of MPV1 and MPV2 VP2 sequences. VP2 amino acids that conform to the MVP1 GenBank sequence are shaded green, and those within the MPV1 sequences that differ from this are shaded red. One residue, at codon 11, is unique to the parental pool, and is shaded orange. Amino acids that are unique to the parental pool and invariant in the selected virus clones are shaded lilac, and those unique to subsets of the selected viruses are shaded yellow. Residues that are unique to MPV2 are shaded blue. GenBank accession numbers are reported in the Methods.

Comparison of the three consensus VP2 sequences, vMSR, vBNR, and vBSK, present in the founder infectious clones, with the nine MPV1 VP2 sequences currently deposited in GenBank, presents some interesting puzzles, as seen in Figure 6. First, the sequence between VP2 codons 439 and 575 of all three founder clones differ from all other published MPV1 VP2 sequences at eight positions, as shown in Figure 6. The likely explanation for this is the selection of a minor subset of *VP2* genes present in the uncloned parental stock by expansion in the transformed human cell line NB324K, as described in the Methods section. Likewise, the sequence between codons 8 and 70 for all but one of the 12 *VP2* genes cloned from the initial stock, represented by MPV1-vMSR in Figure 6, contain five amino acid residues, at codons 11, 12, 54, 57, and 70, that do not appear in any of the nine MPV1 VP2 sequences in published GenBank. As described above, these changes are heavily selected against by serial passage in B16F10, being replaced in most of the MPV1p5 clones by sequences common to all of the other MPV1 strains.

The possible origin of these five apparently unique N-terminal residues in the unselected stock, and indeed, six of the C-terminal residues that are invariant, becomes clearer when the two MPV2 VP2 sequences deposited in GenBank are added to Figure 6. The original GenBank sequences for MPV1 and MPV2 VP2 molecules differ at 65 codon positions, 44 of which are conserved in all nine MPV1 VP2 sequences in the database. These residues presumably contribute to the definition of MPV1 and MPV2 as separate serotypes, and are omitted from the analysis. Only the 27 codons that differ at one or more positions across the twelve aligned MPV1 VP2 sequences are displayed in Figure 6. This shows that six of these residues are unique to MPV2 VP2, while many of the variations within the MPV1 isolates appear to be due to recombination with MPV2, presumably while both are circulating in mice. This is perhaps not surprising, since the study from the University of Louisiana that provided four of the MPV1, and one of the MPV2 VP2 sequences shown in Figure 6, analyzed viruses isolated from serial outbreaks in animal facilities on the same campus [57], and show evidence of hybridization between the two serotypes, as can be seen in the figure. Epidemiological studies across multiple laboratory mouse colonies [58] indicate that MPV2 infections occur at about one-fourth of the frequency of MPV1 infections, suggesting that even in such controlled environments, the possibility of co-infections might be significant.

As shown in Figure 6, four N-terminal residues and six C-terminal residues in MPV1-vMSR that are not found in any MPV1 VP2 sequence are present in both MPV2 VP2 sequences. S8 is shared between one MPV1 and both MPV2 VP2 molecules, while N12 is shared by the consensus starting population, and the original MPV2 VP2, but no others. R11 is unique to the clones isolated in this study, being present in 11 of 12 clones from the starting stock, and half of the selected viruses, suggesting that it arose and spread in the evolution of the starting stock, but was weakly selected against by growth in B16F10 cells. S439 and M461 again appear to be unique to the clones isolated in this study, but are not selected against by propagation in B16F10 cells. Thus, the starting population appears to comprise complex recombinants between MPV1 and MPV2, whose N-terminal MPV2-related sequences are selected against by propagation in B16F10 cells, whereas their C-terminal MPV2-related sequences either are not selected against, or no alternative MPV1 sequences were present in the initial stock that could be selected positively.

While six out of the eight selected changes in VP2 appear to pre-exist in the parental population, and are thus likely not the result of spontaneous mutation during the serial passage, the mutations at positions 348 and 349 do appear to have arisen, and have been selected for, during extended growth in B16F10 cells. They are unique to the selected clones and are not found in any other MPV1 VP2 sequence. Indeed, they occur at codons that are conserved between all of the reported sequences of both MPV1 and MPV2. The observations that they appear to be mutually exclusive, are located next to one another, and each enhance B16F10 infection, strongly suggests that they act through the same mechanism, strengthening the hypothesis that novel mutations that lead to enhanced growth in a particular host cell can arise spontaneously within the virus population.

### 4.2. Localizing the Enhancing Changes within the Capsid Shell

**Figure 7:**
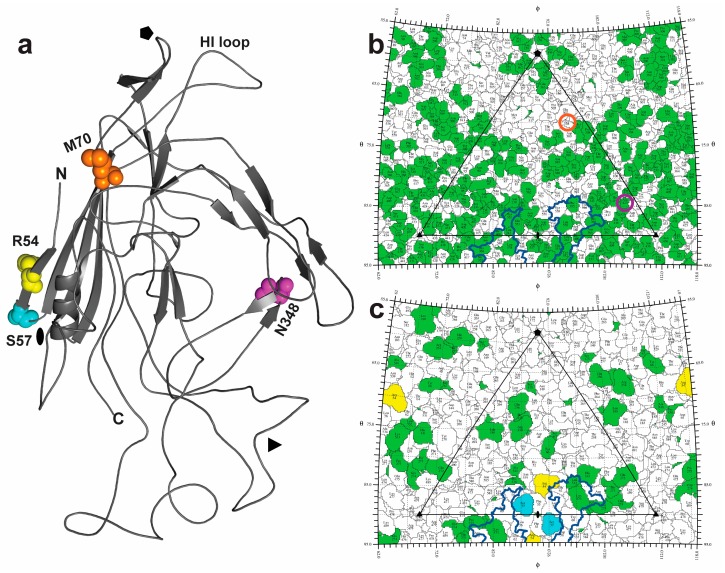
Mapping the melanomatropic determinants of MPV1p5 clone Bv1 VP2 monomer. Panel (**a**) shows a 3D model of the predicted MPV1p5-BV1 VP2 structure. A VP2 monomer shown in cartoon representation with amino acid residues that differ between MPV1p5-Bv1 and MPV1-Mv1 are labeled, and highlighted as spheres: N348 in magenta, M70 in orange, S57 in cyan, and R54 in yellow. The approximate icosahedral symmetry axes are indicated; 2-fold (oval), 3-fold (triangle), and 5-fold (pentagon). N: N-terminus. C: C-terminus. Panels (**b**,**c**) show 2D “roadmaps” of the capsid exterior and interior surfaces, respectively. The large triangle delineates the viral asymmetric unit with the icosahedral symmetry axes that are indicated as in (**a**). The previously identified sialic acid binding site for MVMp is mapped onto the MPV1p5-Bv1 capsid, and outlined in navy blue. Residues that differ between MPV1p5-Bv1 and MVMp are colored in green. Bv1 vs Mv1 amino acid differences are colored as in (**a**) for residues N348 and M70 (buried within the capsid, not visible) as well as, S57 and R54 R54, S57 (capsid interior). As identified in (**b**), N348 is located ~20 Å beneath the capsid surface (positioned below residue 423, which is circled in magenta), and M70 is ~9 Å below residue 508, circled in orange.

The 3D structure of MPV1 is currently not available, thus, a homology model was constructed based on the MVMp VP2 structure (Figure 7a), and from this, residues predicted to be solvent-exposed on the outer and inner surfaces of the capsid were visualized (as shown in Figure 7b,c, respectively). MVMp VP2 was chosen for this modeling, because it both has a very well-characterized structure [46,59], and is >80% related in amino acid sequence to the VP2 of MPV1.

The changes at codons 8, 11, and 12 occur within the dynamic N-terminal segment of VP2, for which the structure has not been determined. While this VP region is sequestered within newly-assembled empty capsids, it is extruded through a pore at the five-fold symmetry axis during the packaging of progeny genomes [55]. This region deploys a nuclear export signal (NES) that is important for transport of DNA-containing virions into the cytoplasm in some cell types [60,61,62]. The domain contains potential serine phosphorylation sites, two of which, S8P and N12S, are reciprocally affected by the selected mutations. In MVM, these sites are variably phosphorylated in different host cells, and appear to affect the function of the NES [63]. Since this region appears to function predominantly in packaging and exit of progeny virions [60,61,62], it is difficult to envision how these late processes could affect the entry and early initiation steps of infection. However, the VP2 N-terminal sequences are cleaved off incoming particles in the endosomal compartment, leading to a conformational change that allows the deployment of the VP1sr, again through the five-fold pore [64,65,66]. This 142 amino acid N-terminal extension of VP1 contains an essential phospholipase 2A (PLA2) enzymatic core required for breaching the endosomal membrane, allowing the virion to enter the cytosol [66,67]. Conceivably, some combination of the three VP2 N-terminal mutations might affect the temporal and/or spatial properties of the VP2 cleavage process necessary for VP1sr deployment, or influence the function of the VP1sr itself.

While the changes at VP2 positions 54 and 57 would be expected to be close to one another in the virion shell, as would 348 and 349, it was surprising that the residue pairs and the change at position 70, are not predicted to cluster in the 3D structure shown in Figure 7a. More puzzling, none of them are predicted to be on the outer capsid surface. The buried I70M change introduces a larger hydrophobic residue within a hydrophobic environment, which would interact with five-fold symmetry related monomers, and is located at the base of the HI loop, thus, might play a role in controlling protein and/or genome extrusion through the five-fold pore. The S348N change is also buried at a site located beneath the three-fold protrusions. N348 has a longer polar side-chain than S348, and could interact with the main chain of G351 within the same monomer. N348 is predicted to interact with R407 and the main chain at residue G404 of a symmetry related VP monomer. While mutation at this residue is therefore predicted to alter several structural interactions, how this change, or the reciprocal switch between lysine and arginine at its neighboring position, might have similar effects on the initiation of infection in B16F10, remains enigmatic.

The model does, however, predict that the residues at positions 54 and 57 are exposed at the inner surface under the two-fold axis. In AAV2, the region surrounding the two-fold axis plays an important role in capsid trafficking events, and in viral gene transcription [68]. Within the current VP2 model, R54 (for Bv1) and K54 (the homologous residue on Mv1) are predicted to make intramolecular interactions at the two-fold axis with E62, which is involved in DNA stabilizing interactions in MVMp. The side chain undergoes a conformational change following genome packaging, suggesting that the mutation here might promote a subtle difference in uncoating kinetics, which could affect the onset of gene expression. These residues lie under the deep indentation that forms part of the glycan binding site, a region to which most mutations affecting tissue tropism have been mapped for variants of MVM [46,59,69,70,71]. Thus, these changes could potentiate, in B16F10 cells, the currently unknown step(s) catalyzed by the crucial virion–glycan interaction.
